# Catabolism of Glucosinolates into Nitriles Revealed by RNA Sequencing of *Arabidopsis thaliana* Seedlings after Non-Thermal Plasma-Seed Treatment

**DOI:** 10.3390/life12111822

**Published:** 2022-11-08

**Authors:** Alexandra Waskow, Anthony Guihur, Alan Howling, Ivo Furno

**Affiliations:** 1 Swiss Plasma Center (SPC), École Polytechnique Fédérale de Lausanne (EPFL), CH-1015 Lausanne, Switzerland; 2 Department of Plant Molecular Biology, Faculty of Biology and Medicine, University of Lausanne (UNIL), CH-1015 Lausanne, Switzerland

**Keywords:** non-thermal plasma, plant defense, glucosinolates, nitriles, RNA sequencing, *Arabidopsis thaliana*

## Abstract

Non-thermal plasma-seed treatments could be an environmentally friendly method to modulate plant properties. Since it remains unclear how plasmas affect seeds, RNA sequencing was used here to analyze gene transcription changes in 7-day-old *Arabidopsis thaliana* (L.) Heynh. seedlings grown from surface dielectric barrier discharge plasma-treated seeds. In a previous study, seeds were analyzed 6 days after plasma exposure and a plant stress and defense response was observed. Here, we performed a pathway analysis on differentially expressed genes and our results revealed again an increased expression of plant stress and defense, specifically glucosinolate pathway-related compounds. The main difference was that a different part of the plant defense response changed at 7 days, which was not previously observed at 6 days. With a 24-h delayed extraction time point, the glucosinolates were selectively broken down into nitriles among all of the glucosinolates catabolic products. Although information about nitriles is limited, it protects plants against biotic stresses and has variable toxicity depending on the interacting organism. More work needs to be performed to better understand which plasma seed treatment parameters affect plant defense; however, these preliminary findings suggest that an optimized plasma treatment could be used to elicit a plant defense response.

## 1. Introduction

The demand in agriculture to minimize or replace current chemical practices has been increasing in recent years and now, biologicals, soil health, and traditional farming practices are gaining traction. Among these approaches, investigations into cold, non-thermal plasma applications on seeds and plants are increasing.

There are a variety of stressors which can elicit a plant defense response, such as heat, chemical, or mechanical stress, and now, the potential of plasma is being explored as a non-toxic, soft chemical treatment. Plasma could potentially avoid additional mechanical and heat damage and due to its multiple components and synergies, it can potentially trigger unique defense responses and outcomes. In theory, an optimized plasma treatment should not produce any toxic residues, and to date, multiple studies have shown that plasma treatments can support germination, growth, disease and stress resistance, delay senescence, and improve crop yield [1,2,3,4,5,6,7].

Plasma is produced when a gas is ionized; it is a combination of UV photons, electric field, electrons, ions, heat, and reactive oxygen and nitrogen species (RONS). Biological applications of non-thermal plasmas are possible because a high-temperature chemistry can be attained at a low gas temperature [1]. There are multiple plasma device configurations but the most common are, by far, the Dielectric barrier discharges (DBDs) at atmospheric pressure. The dielectric layer, unique to this configuration, is used as an insulation barrier to prevent sparking which can eventually lead to arcing at high voltages. Moreover, treatment time and duty cycle are a few examples of variables which can be adjusted to ignite a plasma at a sufficiently low gas temperature, a requirement for biological substrates sensitive to heat, such as seeds.

As cited previously, successful results have been obtained globally, yet there are no clear guidelines which outline the relevant plasma treatment parameters for plasma-induced plant effects. Moreover, due to the limited body of knowledge, these changes are currently unpredictable. Novel information concerning the mechanisms can be discovered by analyzing changes in gene expression, methylation patterns, or protein expression in plants grown from plasma-treated seeds using high throughput methods [8,9,10,11]. Among these methods, studies have mostly resorted to using a quantitative PCR (qPCR) to measure the expression of specific genes of interest, and more recently micro-arrays or RNA sequencing (RNA-seq), although the latter is currently limited [12,13,14,15,16,17,18,19,20,21,22,23,24,25,26,27,28,29,30,31].

Based on our previous studies [32,33], we observed accelerated germination by modifying the plasma treatment times and voltage values. Here, we used RNA sequencing to study the mechanisms behind this plasma-induced phenotype, accelerated germination. *Arabidopsis thaliana* (L.) Heynh. seeds were treated with a dry air plasma, which ignites at the edges of high voltage electrodes in a surface dielectric barrier discharge (SDBD). *A. thaliana* is a plant model organism with an entirely sequenced genome and therefore, it is more feasible to probe the underlying mechanisms and effects of plasma–plant interactions. We first decided to analyze whole seedlings to capture a global overview of the main processes and to avoid influencing the results with additional mechanical stress as a result of separating and isolating different tissue types. However, future experiments should explore tissue-specific changes by analyzing the roots and shoots separately. Furthermore, dry seeds with a low moisture content of 7.66% were used in this study since moisture can influence the plasma seed treatment results [34].

This study is a follow-up of our previous study. RNA sequencing was previously used to analyze 6-day-old seedlings from plasma-treated seeds using two different plasma treatment times of 60 s and 80 s at 7.75 kV ± 3% [32]. Here, RNA sequencing was used once again as a pioneering, preliminary study with similar plasma conditions using a 60 s treatment time at 7.75 kV ± 3% except with a different sampling time point: 7-day-old seedlings [33]. Although we compared our two transcriptome datasets, we are mindful of the minor, albeit relevant, differences. The plasma seed treatments across both studies were performed on the same day and grown in parallel. Furthermore, the same plasma treatment time of 60 s was used again. Minor differences mainly arose from the different sampling time points and voltage values (inherent to plasma treatments). Although 8 kV was measured in the previous time series study, we assumed that 7.5 kV would produce a similar plasma since the voltage was 7.75 kV ± 3%. These two voltages differ only by 6%, which is within the experimental error of the voltage supply to the DBD electrodes.

Specifically in this paper, 6-day-old and 7-day-old untreated, control seedlings were first analyzed and compared. This was followed by a comparison between the untreated, control seedlings at days 6 and 7 with the corresponding plasma-treated seedlings to determine whether the changes in gene expression were due to the plant age or plasma treatment. Finally, we concluded with the differentially expressed genes and pathways of 7-day-old plasma-treated seedlings relative to the untreated 7-day-old seedlings. Increased transcription of glucosinolate-related enzymes such as two genes encoding myrosinases (AT1G51470, AT1G47600) were observed again, suggesting the breakdown of glucosinolates. However, the most striking finding from this study was that the glucosinolates were specifically broken down into nitriles, as indicated by the increased expression of a nitrile specifier protein (AT3G16390), which seems to promote only the production of simple nitriles (Table S3). To our knowledge, this has not been previously reported in the literature for plasma agriculture.

## 2. Materials and Methods

### 2.1. Seed Material

*Arabidopsis thaliana* Col-0 seeds were cultivated at the Department of Plant Molecular Biology at the University of Lausanne. They were grown in a plant chamber room and harvested in May 2019. A thermogravimetric analysis (TGA 4000, Perkin Elmer, Waltham, MA, USA) was completed to verify the low moisture content of the seeds, which was 7.66%. These seeds were stored in Eppendorf or Falcon tubes and kept at room temperature in the dark until used for experiments [32,33]. At the time of the experiments performed in this study, the seeds were 18–20 months old.

### 2.2. Surface Dielectric Barrier Discharge Description

The materials and design of the SDBD device (Sihon Electronics) used alumina as a dielectric and a printed striped pattern for the high voltage electrodes. The SDBD was housed in a closed stainless steel reactor chamber, 18 cm diameter and 11 cm high. For the plasma-seed treatment, the seeds were situated underneath the SDBD device, and were resting on Teflon cylinders approximately 3.7 mm away from the plasma. The seeds were not overlapping. Each individual *Arabidopsis* seed had an area of approximately 0.1 mm^2^ [35]. The seed-plasma treatment was static. Additional details can be found in previous studies [32,33].

### 2.3. RNA Isolation, Library Construction and RNA Sequencing

After the seeds were treated with plasma, they were sown on water agar plates (within hours). The 48-h time point after sowing was used to measure the germination rate. The samples were incubated for another 5 days. After 7 days from the time of sowing, the total RNA (up to 100 mg) was extracted from three biological replicates using a lysing kit with 1.4 mm zirconium beads in 0.5 mL tubes in a Precellys machine (Bertin, Montingy-le-Bretonneux, France). A custom program in Precellys was used entirely at 4 °C as follows: 30 s at 6000 rpm, 10 s at 0 rpm (break), and 30 s at 6000 rpm. To isolate the RNA, InnuPREP Plant RNA kit (Analytic Jena, Jena, Germany) was used and the extracted RNA was quantified using a nanodrop (DS-11 Microvolume Spectrophotometer).

The RNA quality was determined using a Fragment Analyzer (Agilent Technologies, Santa Clara, CA, USA) and all samples used in this study had an RNA quality number (RQN) above 8.3. The Lausanne Genomic Technologies Facility at the University of Lausanne prepared the library and the RNA sequencing (https://www.unil.ch/gtf, accessed on 15 June 2021). For the RNA-seq libraries, 400 ng of total RNA was used in combination with the Illumina TruSeq Stranded mRNA reagents (Illumina) using a unique dual indexing strategy, and following the official protocol automated on a Sciclone liquid handling robot (PerkinElmer, Waltham, MA, USA). A fluorimetric method (QubIT, Life Technologies, Carlsbad, CA, USA) was used to quantify the libraries and a Fragment Analyzer determined the quality (Agilent Technologies, Santa Clara, CA, USA).

Cluster generation was performed with 2 nM of an equimolar pool from the resulting libraries using the Illumina HiSeq 3000/4000 SR Cluster Kit reagents. It was then sequenced on the Illumina HiSeq 4000 SR platform (single end) using HiSeq 3000/4000 SBS Kit reagents for 150 cycles (single end). The sequencing data were demultiplexed using the bcl2fastq2 Conversion Software (version 2.20, Illumina, San Diego, CA, USA). This produced 31–37 million of 150 bp long single-end reads for each library independently, which were then sequenced (Table S1).

Quality control (phred score > 20) and adapter trimming with FastQC (0.11.976), and BBDuk were performed on the raw reads. Any matches to ribosomal RNA were eliminated with fastq_screen (v. 0.9.3). The *Arabidopsis* reference genome sequence (Araport11) and the default parameters in STAR v2.7.5 were used for read alignment. The count matrix was generated with FeatureCounts v1.6.2 in order to calculate gene expression values as raw read counts. This was used to obtain RPKM to make heatmaps with an in-house script.

The gene expression profiles are of 7-day-old seedlings (NCBI project number PRJNA800224). The seeds were treated with a 7.5 kV plasma for 60 s and grown until the 7th day in the same agar plate under continuous light to reduce biological variability. A pool of 30 seedlings represents one biological replicate. An average of ~47 million raw reads of 150 base pairs (bp) were produced. After filtering, ~46 million clean reads per library were retained (Figure S1, Table S1) and ~96% were mapped to the *A. thaliana* reference genome (Table S2).

DESeq2 package from R software v1.30.1 [36] after rlog transformation and Wald test with the *p*-value adjusted using the Benjamini and Hochberg method (FDR), were used to analyze count read values in order to identify the differentially expressed genes (DEGs) between untreated and treated samples. ShinyGO v.0.76 software was used to find GO categories of differentially expressed genes with *p*-value cut-off set at <0.05 [37]. The results were based on customized background genes from our RNA-seq, which yield more accurate results for enrichment analysis [38]. The transcriptome data are available in NCBI Bioproject Code: PRJNA800224 [32].

## 3. Results

### 3.1. Global RNA-seq Analysis of Young Seedlings Grown from Plasma-Treated Seeds

**Figure 1:**
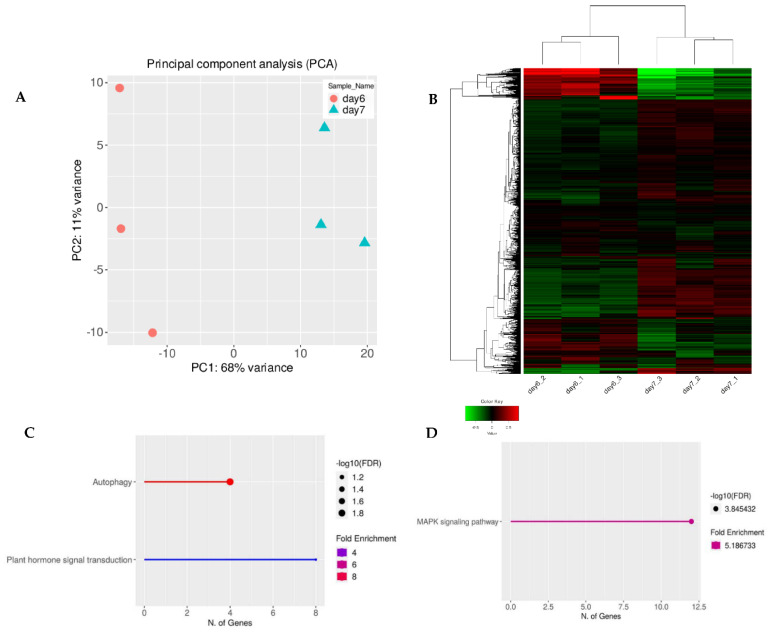
(**A**) Normalized gene expression values used in Principal Component Analysis (PCA) for 6-day-old and 7-day-old samples. The two components, PC1 and PC2, are shown on the X- and Y-axes with 68% and 11% variance, respectively. Orange circles represent triplicates of 6-day-old untreated *A. thaliana* seeds grown into seedlings and blue triangles represent the same as the orange circles except that they were 7-day-old seedlings. Each point in the plot represents a biological replicate, representing 30 seedlings, with a total of 6 biological replicates in the plot. (**B**) The full transcriptome for 6-day-old and 7-day-old untreated seedlings represented as a heat map (Z-scaled reads per kilobase of exon per million reads mapped (RPKM)). The relative expression profile of the top 2000 variable genes were selected based on the lowest standard deviation using Euclidean distance and are shown as hierarchical clustering. The columns represent individual samples, and the rows represent genes. The color scale represents the relative read count of genes: green indicates low relative read counts; red indicates high relative read counts; black indicates zero (no change). (**C**) Pathway enrichment analysis of upregulated genes using KEGG category. (**D**) Pathway enrichment analysis of downregulated genes using KEGG category. Significant differences between untreated 6-day-old and 7-day-old untreated, control seedlings were due to plant development.

**Figure 2:**
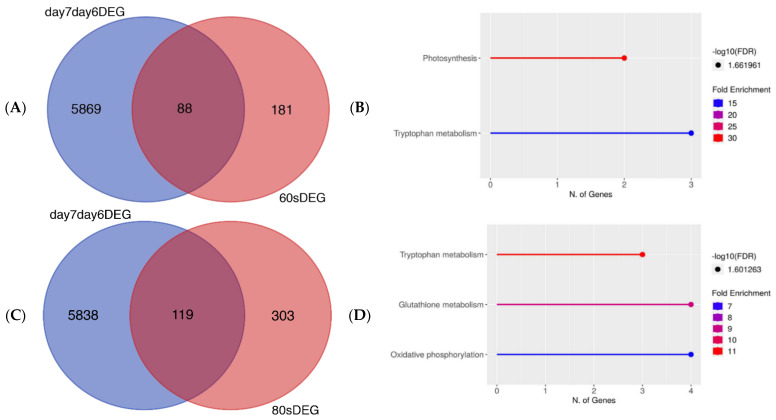
(**A**) Venn diagram showing number of DEGs which overlapped or differed between DEGs in 60 s plasma treatment triplicates from [32] (red) and DEGs shared between 6-day-old and 7-day-old untreated seedlings (blue). (**B**) Pathway enrichment analysis using KEGG for DEGs in (**A**). (**C**) Venn diagram showing number of DEGs which overlapped or differed between 80 s plasma treatment triplicates from [32] (red) compared to DEGs shared between 6-day-old and 7-day-old untreated seedlings (blue). (**D**) Pathway enrichment analysis using KEGG for genes in (**C**). Venn diagrams demonstrate very few genes in common between untreated and plasma-treated seeds grown into seedlings. Related genes are involved in primary metabolism and growth.

**Figure 3:**
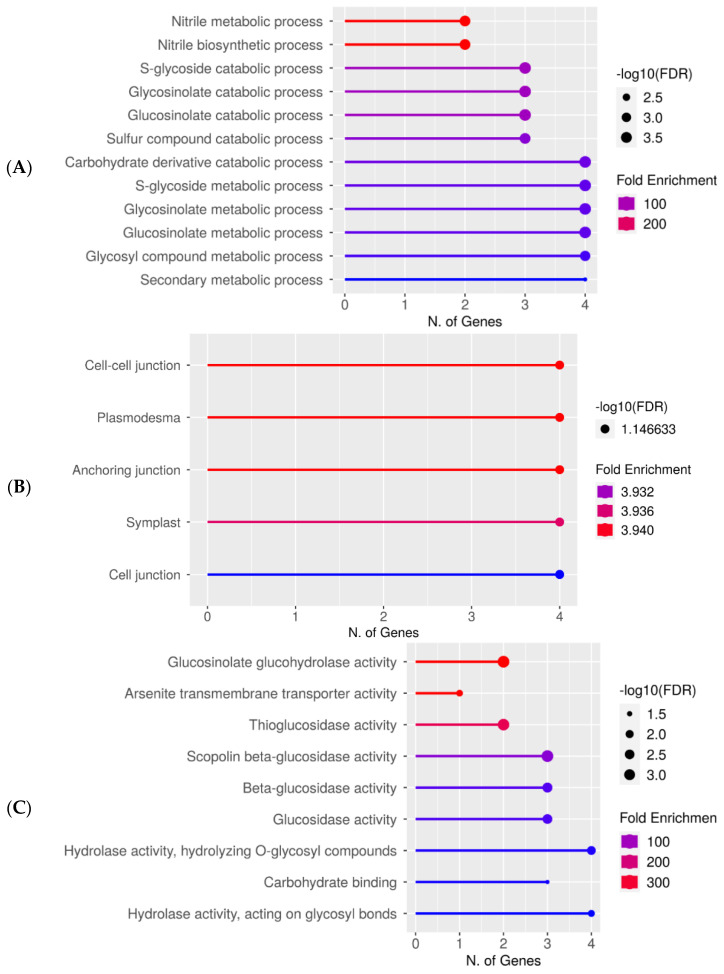
Upregulated gene enrichment analysis after 60 s plasma treatment at 7.5 kV. GO fold enrichment, significance (FDR in log10), and number of genes in each pathway are given in the lollipop diagrams in the following categories: (**A**) biological process, (**B**) cellular component, and (**C**) molecular function.

**Figure 4:**
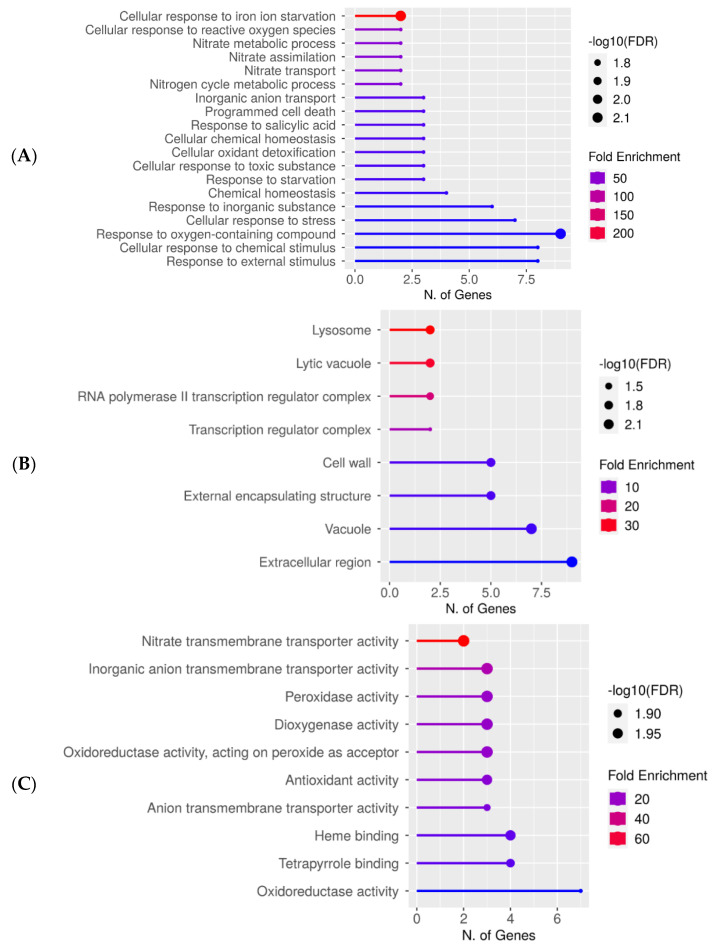
Downregulated gene enrichment analysis after 60 s plasma treatment at 7.5 kV. GO fold enrichment, significance (FDR in log10), and number of genes in each pathway are given in the lollipop diagrams in the following categories: (**A**) biological process, (**B**) cellular component, and (**C**) molecular function.

The normalized gene expression values were used in the Principal Component Analysis (PCA). There was clustering among the three replicates of the untreated 6-day-old seedlings and untreated 7-day-old seedlings (Figure 1 and Figure 2). However, there was little clustering among the three replicates of the 7-day-old plasma-treated seedlings and the untreated 7-day-old seedlings. Nevertheless, there was clustering when using two replicates. Therefore, for further data analysis, one replicate of the control and plasma-treated sample was removed to reduce the variability (Figure 3, Figure 4 and Figures S2–S4). In total, 75% of the variance was explained by the first two principal components (59% by PC1 and 26% by PC2). From the 32,833 genes across four samples, 21,168 genes passed the selected threshold; each biological replicate had more than two reads (see Methods for more details).

A false discovery rate (FDR) < 0.15 and a log2foldchange (FC) > 1 were used for the analysis. There were 27 upregulated genes, and 29 downregulated genes for a total of 56 differentially expressed genes (DEGs). It should be noted that only a few enriched genes were identified in this study and therefore, our statements are made tentatively. The main focus of this manuscript is to highlight the similarities and differences between 6-day-old and 7-day-old seedlings treated with a similar plasma. Both the 6-day-old and 7-day-old seedlings had transcriptional changes related to the glucosinolate metabolism, yet only the transcription of a nitrile specifier protein was detected in the 7-day-old seedlings.

### 3.2. Comparison of Gene Expression between 6-Day-Old and 7-Day-Old Untreated, Control Seedlings

To ensure that the observed changes in secondary metabolism were caused by the plasma treatment and not plant age, we cross-referenced our data and analyzed the gene expression profiles for 6-day-old untreated, control seedlings (data taken from our previous study) and 7-day-old untreated seedlings (data obtained during this study). PCA analysis and hierarchical heat map clustering revealed significant differences between 6- and 7-day-old seedlings (Figure 1A,B). However, based on pathway enrichment analysis, the main differences were linked to plant development (Figure 1C,D).

### 3.3. Comparison of DEGs between 6-Day-Old and 7-Day-Old Untreated, Control Seedlings to DEGs in Plasma-Treated Seedlings

We then compared the DEGs of untreated, control seedlings and seedlings grown from plasma-treated seeds to check the similarities and differences in the gene expression profiles. To make it possible to produce a Venn diagram, the DEGs from 6-day-old and 7-day-old, untreated seedlings needed to be identified and then compared. By using the changing genes between the 6th and 7th days, a comparison could then be made with the DEGs of plasma-treated seedlings. More details about the DEGs in the 60 s and 80 s plasma-treated samples can be found in a previous study [32]. In Figure 2A,C, there is a minor overlap of significantly DEGs for both days 6 and 7. The untreated and 60 s plasma-treated seedlings showed 88 genes in common between the two conditions; however, there were 5869 DEGs and 181 DEGs in the untreated and plasma-treated samples, respectively. A similar pattern was observed with 80 s plasma-treated seedlings. There were 5838 DEGs and 303 DEGs for untreated and plasma-treated samples, respectively, of which 119 genes overlapped between the two conditions. In both instances, the overlapping genes were related to the primary metabolism (Figure 2B,D), which is involved in growth and development. These genes were found in pathways related to photosynthesis and oxidative phosphorylation, which are known to produce energy. This provided more confidence to ascribe the changes in secondary metabolism to the plasma treatment.

### 3.4. Gene Expression of Plasma-Treated Seeds Grown into 7-Day-Old Seedlings

Gene ontology (GO) analysis of specific DEG groups was used in ShinyGO v0.76 software for the pathway enrichment analysis on two replicates of the 7-day-old seedlings grown from plasma-treated seeds [37]. In Figure 3A–C, the upregulated genes after 60 s plasma treatment at 7.5 kV are organized into biological process, cellular component, and molecular function categories, respectively. The number of genes and fold enrichment in the pathway are shown in the lollipop diagrams, whereas the hierarchical tree clustering is shown in the supplemental section (Figure S3). The individual genes are listed in Table S3. Overall, gene expression increased in the secondary metabolic pathways, mainly for products from the glucosinolate metabolism. Specifically, gene expression in nitrile biosynthesis and metabolism was highly upregulated only in 7-day-old seedlings (Figure 3A, Table S3). Within the cellular component category, components concerning the cell periphery were upregulated and equally had the highest number of upregulated genes (Figure 3B). The upregulated molecular functions were enzymatic reactions related to glucosinolates, glucohydrolase activity, and other enzymes involved in glucosinolate metabolism (Figure 3C).

The lollipop diagrams for the downregulated genes based on two replicates are shown in Figure 4, whereas the hierarchical clustering trees can be found in the supplemental section (Figure S4). The individual genes are listed in Table S3. Overall, gene expression decreased across the diverse pathways related to response to stress or chemical stimulus. Specifically, the cellular responses to iron ion starvation and reactive oxygen species were highly downregulated, whereas responses to the oxygen-containing compound had the highest number of enriched genes (Figure 4A). Within the cellular component category, lysosome and lytic vacuole were the most downregulated and the extracellular region had the highest number of downregulated genes (Figure 4B). The downregulated molecular functions were enzymatic reactions involved in nitrate transmembrane transporter activity or oxidative response (Figure 4C).

## 4. Discussion

### 4.1. Comparison between Our Studies

The aim of our study was to investigate how plasma-seed treatments affect the subsequent seed development on a molecular level. Approximately one-week-old seedlings were used after the root and shoot emergence for the following reasons: first, to ensure that transcriptional changes would be detected and second, because seedlings have increased sensitivity to stress. Limited treatment times and voltages were used to minimize additional stresses such as heat. Moreover, only the two formerly mentioned parameters among five (voltage, time, gas flow rate, plasma-seed gap distance, and frequency) resulted in accelerated germination, which increased our confidence that there would be detectable molecular changes [32,33]. Our previous findings are supported by a study performed by Šerá et al. [39], which showed how the pools of hormones change depending on a short or long plasma treatment time. However, this has not yet been shown using transcriptomics and therefore, this was completed using different plasma treatment times [32], and in this study, using different sampling time points.

The time series study [32] used two plasma treatment times of 60 and 80 s at 8 kV and the RNA was extracted 6 days after plasma-seed treatment. In this study, a single plasma treatment time of 60 s at 7.5 kV was used but the RNA was extracted 24 h later, 7 days after plasma-seed treatment. Previously, we demonstrated that a brief dry synthetic air plasma-seed treatment had a long-term memory effect since it modulated the primary and secondary metabolisms of 6-day-old seedlings. First, the transcription of genes belonging to the phenylpropanoid pathway were upregulated after a 60 s treatment. This pathway is responsible for lignin cell wall reinforcement and the production of antimicrobial compounds, such as phytoalexins. We tentatively interpreted this as a bacterial or fungal plant pathogen defense response. Second, the transcription of genes belonging to the glucosinolate pathway was upregulated after an 80 s treatment. We hypothesized and interpreted this response as a feeding deterrent and thus, as an insect and herbivore defense response. In both instances, it seems that plasma behaved as an oxidative stress and possibly as a wounding. Both the 6-day-old and 7-day-old seedlings had transcriptional changes related to glucosinolate metabolism, yet only the transcription of a nitrile specifier protein was detected only in the 7-day-old seedlings.

Concerning the dataset presented here, we rationalize that the lack of clear clusters between the triplicates of the untreated and plasma-treated samples could be due to the inherent seed variability, plasma-seed treatment variability, or the different sampling time point. For example, fewer DEGs were identified in this study, likely due to the response dampening over time. The latter is possible because this was demonstrated in another study with *Andrographis* where the earliest and latest time points had fewer DEGs [26]. Nevertheless, the results of the data analysis were coherent with previous observations and therefore, they were further analyzed and interpreted.

Prior to the DEG analysis of the 7-day-old seedlings, the gene profiles were compared between the untreated and plasma-treated samples to ensure that the gene expression changes were in fact due to the plasma treatment and not the plant physiology. Indeed, the genes which were common between the two sample types were involved in developmental processes (Figure 1 and Figure 2), indicating that the DEGs were a result of plasma treatment.

Since these changes could be attributed to the plasma treatment, a comparison could then be made more confidently. When comparing the results from this study to our previous study, the gene expression trend in the first study showed a few upregulated genes and vastly more downregulated genes, whereas here, it was an equal ratio of up- and downregulated genes. The list of genes specifically induced after plasma treatment is shown in Table S3. It was initially expected that the upregulation of the phenylpropanoid pathway would be observed again when comparing 60 s at 8 kV to 60 s at 7.5 kV despite the plant age difference. However, the 60 s at 7.5 kV mimicked more closely the 80 s at 8 kV since there was an increased gene expression in glucosinolate-related production and enzymatic activity (Figure 3). Upon further thought, it would be reasonable to observe this since there was an additional 24 h prior to sampling or in other words, extraction. We hypothesize that the phenylpropanoid response shifted towards a glucosinolate response. Perhaps over time, the plant runs through a sequence of pathways, led by gene expression changes, and the same events could be observed with a less intense plasma, with a later sampling time point. In other words, each of these plasma-treatments might have sequentially undergone a phenylpropanoid biosynthesis response, followed by a glucosinolate biosynthesis response, and then nitrile biosynthesis. Depending on the plasma intensity and elapsed time, a different response might have been observed. It is entirely plausible that gene transcription could have changed within 24 h; it is the case that some heat shock proteins change within only 30 min [40].

Regarding the downregulated genes, there were subtle differences in the gene expression of hormones, where only auxin catabolism was observed in the previous study but here, salicylic acid (SA) was observed for the first time (Figure 4A). Auxin was reasonable to observe since aldoxime is a precursor to indole glucosinolates, camalexin, or auxin [41]. It is known that indole glucosinolates are blocked by high levels of auxin and it is likely the same inversely. However, SA is involved in systemic acquired resistance, which would be complementary to the upregulation of the secondary metabolism.

There are similarities between the two studies, which remain with the organelles, especially lysosomes being the most downregulated. The same rationale as before applies again here. The oxidized proteins as a result of plasma treatment could have been cleared before the extraction. Furthermore, oxidation played a role again in eliciting a response since many functions related to oxidation, detoxification, or chemical stress were observed (Figure 4C).

### 4.2. Data Support the Hypothesis about Wounding and Oxidative Stress as a Plant Response to Plasma

If the upregulated genes are analyzed closely, the data supports the proposed hypothesis where plasma could be interpreted as a wounding from an insect or a penetrating fungus or bacterium. We observed here an increase in pathway enrichment for cell wall biogenesis. We hypothesize that this was either related to growth or the plant was repairing damage and reinforcing the cell wall. It remains unknown which plasma components caused these transcriptional responses. To answer this question, it would require a detailed quantification of all the relevant RONS and their spatial distribution as a function of discharge parameters in the presence of the seeds. This would require investigations well beyond the scope of the present paper. However, preliminary studies in this direction have been undertaken, so we speculate that the cascade of transcriptional changes could be due to the diffusion of low concentration short-lived RONS, such as NO, which somehow does not affect the seed surface substantially. Based on our previous findings [27], we detected the presence of NO amongst other species with preliminary LIF studies. However, we observed no changes in the concentrations of carbon, oxygen, nitrogen, or other elements at the seed surface after plasma treatment using XPS (Figure S5). We assume that it may not be necessary for the plasma to interact with the entire seed surface to have changes on a molecular level. It has previously been shown that the seed surface facing the plasma was the only surface to experience any surface changes [1]. If the observed effects occur with only partial exposure of the seed to plasma, then the effects when the whole surface is exposed (for example, in a fluidized bed plasma reactor) can be assumed to be even stronger. In our study, seeds were checked before and after the treatment for any obvious changes in seed positioning and we can confirm that it was a static treatment with no seed movement. Since the seeds were not overlapping and an individual *Arabidopsis* seed is roughly 0.1 mm^2^, probably half of each seed surface (0.05 mm^2^) was in contact with plasma-derived components. Moreover, an indirect treatment using a 3.7 mm plasma-seed gap is unlikely to affect the seed with ions, electrons, or electric fields and thus, this leads us to believe it was RONS diffusion.

Nevertheless, there were transcriptional changes concerning the cell wall; specifically, extensin for cell wall protection (AT1G26240) and chitinase family protein (AT2G43610) were also upregulated, suggesting again cell wall reinforcement and protection against invasion. This could be because mechanical stimulus was detected, which triggers plant defense against wounding since the gene expression of mechanosensitive channel of small conductance-like 9 (AT5G19520) was upregulated. In case this is a response to the plasma-generated RONS, the plasma component could have travelled through an aquaporin, based on the DEGs in this study, which is known to be involved in hydrogen peroxide transport (AT4G19030). Alternatively, it may be due to a few UV photons since a gene involved in DNA repair and toleration (AT3G12610) was upregulated and this would be a typical response to UV.

From this mechanical stimulus, the plant might have responded with cell wall loosening using expansin, which has been mentioned before in other studies and one of these genes (AT5G02260) was upregulated in our dataset. It is interesting to see that the listed effects persisted for 7 days, even though they would be expected to occur shortly after the plasma-seed treatment and with the onset of germination (within the first 48 h in this case). It is often mentioned that abscisic acid decreases and gibberellic acid increases prior to germination and in our dataset, a gene (AT5G15230) which promotes gibberellic acid and exhibits redox activity was upregulated. Since the extraction took place 6 or 7 days after the plasma treatment, the response to the initial stimulus might have evolved into a glucosinolate response, which has been observed in both datasets, specifically with the upregulation of two genes encoding myrosinases (AT1G51470, AT1G47600). The novel aspect of this work was that these glucosinolates were further broken down into nitriles, as indicated by the increased expression of a nitrile specifier protein (AT3G16390), which seems to promote only the production of simple nitriles. There were no major transcriptional changes in the genes involved in thiocyanate formation.

### 4.3. Plasma Defense Activated with Increased Nitrile Synthesis

There are several breakdown products when glucosinolates are in contact with myrosinases, enzymes which are typically stored in different compartments. Once in contact, it results in a defense response with the production of thiocyanates, isocyanates, or nitriles (see [41], Figure 1). It was shown in another study where young mustard greens after plasma treatment had an increase or even doubling of isothiocynates [42,43], which is coherent with the activation of the glucosinolate pathway here.

These processes are regulated by MYB transcription factors, which were not strongly observed in our list, but there was a strong presence of nitrile-related genes. Although the breakdown of glucosinolates into nitriles is not novel [44], to our knowledge, it is the first time that this has been observed after plasma treatment. We cannot be certain that all glucosinolates are broken down into only nitriles without additional metabolic studies, so we propose these dynamic changes based on the obtained RNA-seq data. Considering that there is only one other study plasma-treating *Arabidopsis*, it will become clearer in time whether this speculation is true [27]. It is not yet clear why nitriles are favored over other forms. Ultimately, nitriles can be broken down further into cyanogenic glucosides, so it appears that a very potent response might be elicited from the plant after plasma treatment. In some instances, nitriles are less toxic than isothiocyanates [45]. However, certain organisms have evolved to consume breakdown products through coevolution [46]. In other instances, it can be more toxic to some herbivores or particularly to insects, so it seems to depend on what is attacking the plant [46]. The plant needs to carefully manage its resources, especially for defense responses. It is generally costly for the plant to defend itself, especially at the expense of growth. Therefore, the plant needs to choose when it would be best to mount a response and to which extent. There is a diverse combination of glucosinolate plant responses which are dependent on the concentration and type of glucosinolate, as well as the type of glucosinolate hydrolysis or breakdown products. This intricacy is a reflection of the complex interplay between microbial pathogens, insects, herbivores, and plants. If only glucosinolate biosynthesis is taken into consideration, there are 120 different glucosinolates to date, which is an impressive number to achieve such diversity from just a few amino acids [47]. The variation in glucosinolates is further amplified after glucosinolate breakdown; products can be broken down into a single glucosinolate or into products with diverse physicochemical and biological behavior. Therefore, it is difficult to reach a conclusion about the nitrile synthesis and plasmas without understanding the fundamental biology [48].

## 5. Conclusions

Overall, our findings here support the previously proposed hypothesis: the plant interprets plasma as an oxidative stress as well as a wounding, seemingly tuned to the plasma intensity [32]. Yet this time, another dimension was added by varying a biological parameter (sampling time point) instead of a physical parameter (plasma treatment time). Specifically, what was observed here was that the breakdown of glucosinolates led to the production of nitriles and not isothiocyanates. Although plasma can elicit a plant defense response and assuming that the biosynthesis of particular compounds can be increased and beneficial, it is important to understand at what cost and under which context it would remain so. For example, the biosynthesis of sweeteners increased at the expense of other secondary metabolites [49]. In our study, nitriles were not as poisonous as other glucosinolate breakdown products to certain biota so it may not be problematic but it might be better tolerated than other forms. Therefore, it is very difficult to foresee what effect this would have on plant–biota interactions without multiple bioassays reflecting a more natural environment. Caution should be exercised to not make the plant more susceptible to attacks.

So far, it seems that different pathways can be activated when using different combinations of plants and plasma treatments. For example, plants belonging to the *Brassica* family, such as our studies with *A. thaliana*, can activate the glucosinolate pathway after plasma treatment. However, this is absent in other plant families so other plants such as basil and pea might activate the phenylpropanoid pathway to increase essential oil production or increase lignification, simply due to the plant characteristics rather than the plasma. It thus would be valuable to understand how these changes occur between different plants. In the event that similar pathways are activated, there might be a preference of one pathway over the other. Therefore, one of the next aims should be to understand under which conditions a pathway is activated meanwhile following the genetic changes in parallel [50,51].

Furthermore, it would be important to identify the limit of plasma treatment before it becomes deleterious or activates apoptosis, programmed cell death. In certain contexts, the biosynthesis of particular compounds could be desired and therefore, it might not matter that the plants would die a few days later, given that they are harvested beforehand. However, this would be critical to understand if the plants are grown over the long-term. Therefore, experiments monitoring changes over time after sowing using multiple time points would expedite our understanding. As an example, an extended version of a study performed by the authors of [52] where the authors monitored and observed increased heat shock proteins in the first two days after sowing in plasma-treated corn should certainly be considered when designing experiments.

Lastly, to echo what was stated previously, a multi-omics approach will significantly advance the pace of this research field. Overall, plasma duration was studied before [32], and now, sampling/extraction time has been studied, which are both among the first, pioneering transcriptomics studies performed using *Arabidopsis* and plasma and hopefully, more will be completed in the future. Since there are presently only two *Arabidopsis*-plasma studies [27,33], this study was used to first detect any major changes in gene expression to be able to improve on the experimental design for future studies. Now that we have observed changes primarily in the glucosinolate biosynthesis and breakdown, future experiments will include mass spectrometry metabolomics where the glucosinolate and their breakdown products are extracted and quantified to directly validate these observations at a transcriptomic level. Furthermore, the concentrations of these defense metabolites should be studied indirectly using bioassays with predators, such as caterpillars or fungal pathogens, to determine if these plasma-induced effects do, in fact, protect the plant. In order to improve our general understanding, this will require the investigation of multiple layers, such as genomics, epigenomics, proteomics, and metabolomics. Since the data interpretation from one of these layers cannot be easily transposed to another, the implications remain unpredictable, i.e., gene expression does not always correlate with protein expression. Moreover, genes and proteins are subtly regulated through methylation or phosphorylation, respectively, and therefore, analyzing each and every layer can reveal information about the subtle fine-tuning of plasma treatments. Specifically, it would be interesting to look at the epigenomics and the modification of wrapped DNA since changes in methylation are presently observed but are not yet correlated with the phenotypic changes induced by plasma treatment [53].

In summary, it appears that regardless of minor changes in the plasma-seed experiment, a similar sequence of events might be observed: phenylpropanoid response is triggered first, followed by glucosinolate biosynthesis, and then catabolism into nitriles. Future studies will include more variables in the parameter space, such as multiple sampling time points and variables in the plasma treatment. This will help to determine whether the same response is observed regardless of minor plasma treatment changes or whether minor changes in plasma can trigger exclusively different responses. Nevertheless, our pioneering study brings new facts and clues in the field of a non-thermal plasma treatment with possible agronomic interests, which could serve as the foundation for future studies to build upon.

## Data Availability

The data presented in this study are available in this article, supplementary material and the transcriptome data are available in NCBI Bioproject Code: PRJNA800224.

## Supplementary Materials

The following supporting information can be downloaded at: https://www.mdpi.com/article/10.3390/life12111822/s1. Figure S1: Quality control for 7-day-old, untreated, control seedlings and 60 s, 7.5 kV plasma-treated seeds grown into seedlings (A) Genes retained in each sample (B) normalization of samples (C) density plot to demonstrate that profiles are similar to proceed with analysis.; Figure S2: (Left) Principal Component Analysis (PCA) conducted on the normalized gene expression values of the 7-day-old samples at 7.5 kV. X- and Y-axes show PC1 and PC2, respectively, with the amount of variance contained in each component, which 59% and 26%. Each point in the plot represents a biological replicate, representing 30 seedlings, with a total of 4 biological replicates in the plot. Symbols of the same colors are replicates of the same experimental group where orange represents the control which are untreated A.thaliana seeds grown into seedlings and blue represents 7.5 kV plasma-treated A.thaliana seeds grown into seedlings. (Right) Heat map of the expression patterns (Z-scaled reads per kilobase of exon per million reads mapped (RPKM)) of the full transcriptome for 7-day-old samples at 7.5 kV. Hierarchical clustering of the relative expression profile of the top 2000 variable genes selected based on the lowest standard deviation using Euclidean distance. Individual samples are shown in columns, and genes in rows. The left vertical axis shows clusters of genes. The color scale represents the relative read count of genes: green indicates low relative read counts; red indicates high relative read counts; black indicates zero (no change); Figure S3: A hierarchical clustering tree for the upregulated genes after 7.5 kV plasma treatment in 7-day-old seedlings, summarizing the correlation among significant pathways within GO categories (A) biological process (B) cellular component (C) molecular function. Pathways with many shared genes are clustered together. The blue, bigger dots followed by the FDR values indicate more significant P-values.; Figure S4: A hierarchical clustering tree for the downregulated genes after 7.5 kV plasma treatment in 7-day-old seedlings, summarizing the correlation among significant pathways within GO categories (A) biological process (B) cellular component, and (C) molecular function. Pathways with many shared genes are clustered together. The blue, bigger dots followed by the FDR values indicate more significant P-values.; Figure S5: X-ray photoelectron spectroscopy (XPS) analysis with atomic concentration table of carbon, nitrogen, oxygen, silicon, and potassium in untreated, and plasma-treated Arabidopsis seeds. From top to bottom, plasma treatment used different times, voltages, and plasma-seed gap distances.; Table S1: Number of NGS-RNA-seq reads before and after quality check on the raw sequencing data for 7-day-old seedlings treated with 7.5 kV plasma.; Table S2: Number of clean reads mapped against A. thaliana genome for 7-day-old seedlings treated with 7.5 kV plasma.; Table S3: List of DEGs using a 60 s, 7.5 kV plasma treatment with (left) upregulated genes and (right) downregulated genes with their corresponding fold change.
