# Supplementary material for: Catabolism of Glucosinolates into Nitriles Revealed by RNA Sequencing of Arabidopsis thaliana Seedlings after Non-Thermal Plasma-Seed Treatment
Source: Life (Basel). 2022 Nov 8;12(11):1822. doi: 10.3390/life12111822 (PMC9696580; doi:10.3390/life12111822)
Supplement: Supplementary file 1 [file life-12-01822-s001.zip › life-1985635-supplementary.pdf]

Supplemental materials

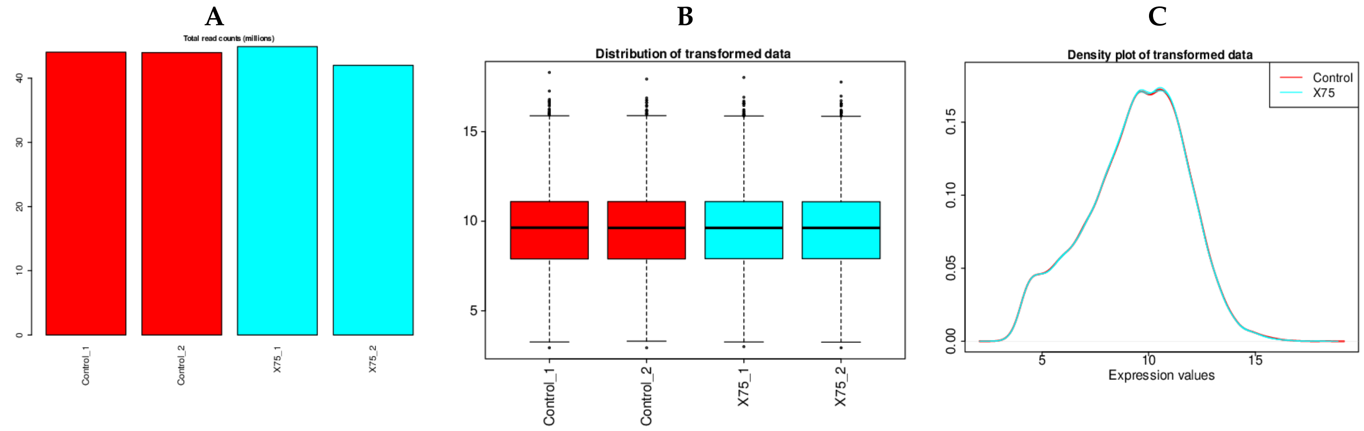

Figure S1. Quality control for 7-day-old, untreated, control seedlings and 60 s, 7.5 kV plasma-treated seeds grown into seedlings **(A)** Genes retained in each sample **(B)** normalization of samples **(C)** density plot to demonstrate that profiles are similar to proceed with analysis.

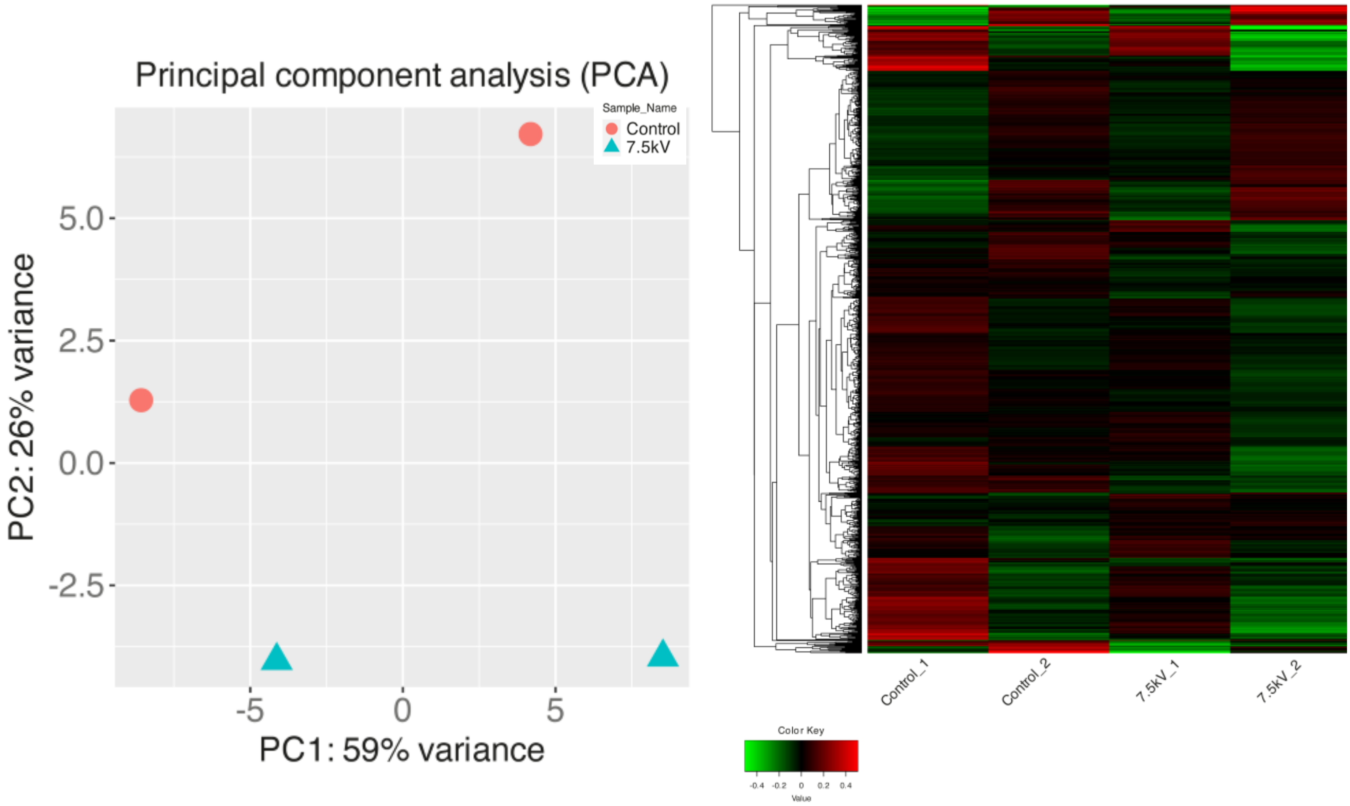

Figure S2. **(Left)** Principal Component Analysis (PCA) conducted on the normalized gene expression values of the 7-day-old samples at 7.5 kV. X- and Y-axes show PC1 and PC2, respectively, with the amount of variance contained in each component, which 59% and 26%. Each point in the plot represents a biological replicate, representing 30 seedlings, with a total of 4 biological replicates in the plot. Symbols of the same colors are replicates of the same experimental group where orange represents the control which are untreated *A.thaliana* seeds grown into seedlings and blue represents 7.5 kV plasma-treated *A.thaliana* seeds grown into seedlings. **(Right)** Heat map of the expression patterns (Z-scaled reads per kilobase of exon per million reads mapped (RPKM)) of the full transcriptome for 7-day-old samples at 7.5 kV. Hierarchical clustering of the relative expression profile of the top 2000 variable genes selected based on the lowest standard deviation using Euclidean distance. Individual samples are shown in columns, and genes in rows. The left vertical axis shows clusters of genes. The color scale represents the relative read count of genes: green indicates low relative read counts; red indicates high relative read counts; black indicates zero (no change).

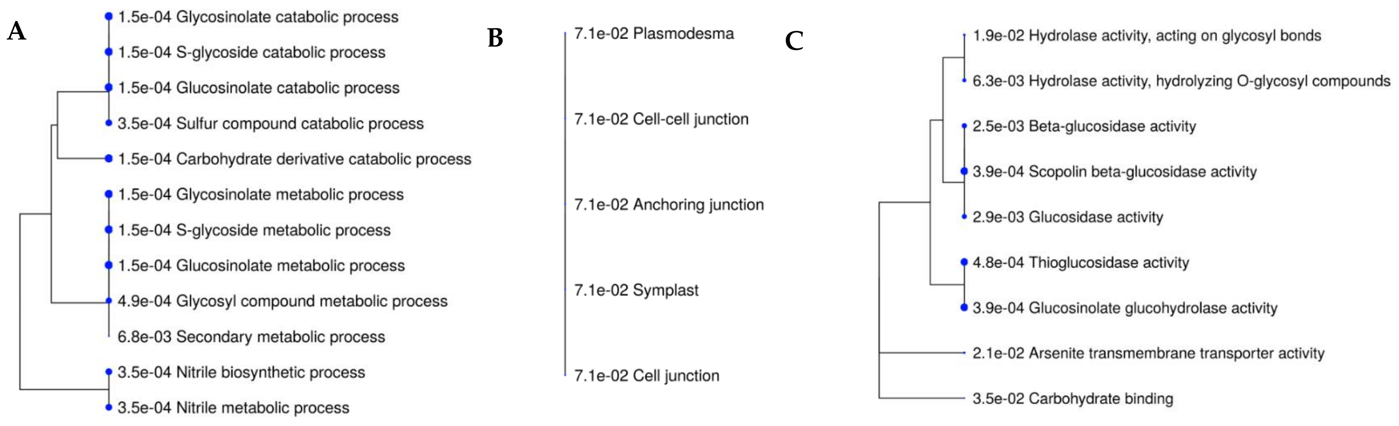

Figure S3. A hierarchical clustering tree for the upregulated genes after 7.5 kV plasma treatment in 7-day-old seedlings, summarizing the correlation among significant pathways within GO categories **(A)** biological process **(B)** cellular component **(C)** molecular function. Pathways with many shared genes are clustered together. The blue, bigger dots followed by the FDR values indicate more significant P-values.

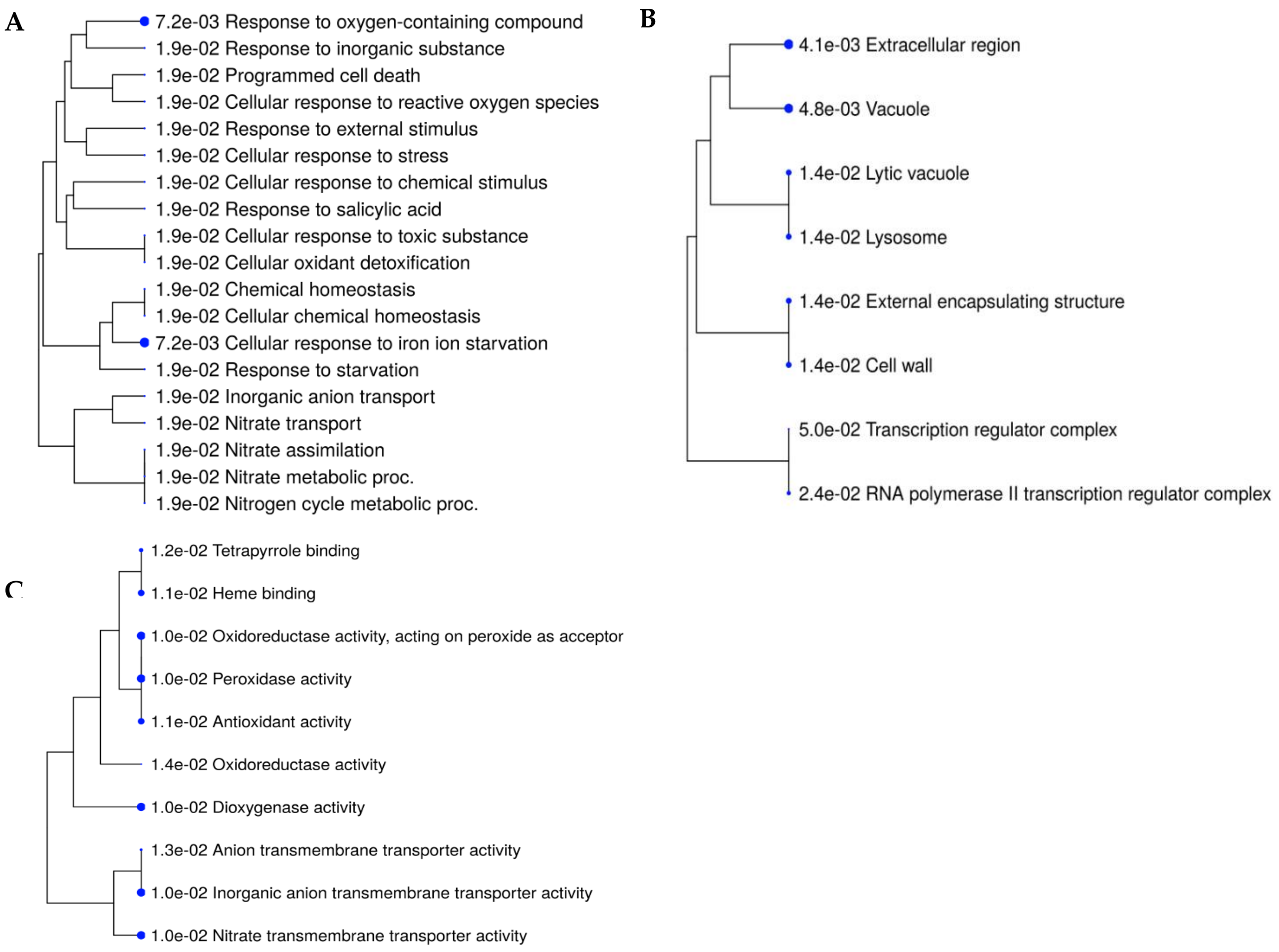

Figure S4. A hierarchical clustering tree for the downregulated genes after 7.5 kV plasma treatment in 7-day-old seedlings, summarizing the correlation among significant pathways within GO categories **(A)** biological process **(B)** cellular component, and **(C)** molecular function. Pathways with many shared genes are clustered together. The blue, bigger dots followed by the FDR values indicate more significant P-values.

AC + stripes	C1s [%]	N1s [%]	O1s [%]	Si2p [%]	K2p [%]
untreated	89.21	0.15	8.06	1.54	0.98
20 s	91.20	0.35	7.53	0.87	0.00
60 s	88.13	0.60	9.11	1.21	0.95
80 s	89.3	0.32	8.19	1.20	0.81

AC + stripes	C1s [%]	N1s [%]	O1s [%]	Si2p [%]	K2p [%]
untreated	89.01	0.00	8.96	1.71	0.11
6.5 kV	88.87	0.76	8.96	1.14	0.10
7.5 kV	87.23	0.23	10.24	1.94	0.09
8.5 kV	89.22	0.21	9.21	1.20	0.11
9.5 kV	89.42	0.35	8.34	1.65	0.10

AC stripes	C1s [%]	N1s [%]	O1s [%]	Si2p [%]	K2p [%]
untreated	89.24	0.67	8.28	1.70	0.11
4.33 mm	86.54	0.00	10.93	2.12	0.10
3.7 mm	87.51	0.44	9.80	2.04	0.12
3 mm	87.31	0.00	10.51	1.88	0.09

Figure S5. X-ray photoelectron spectroscopy (XPS) analysis with atomic concentration table of carbon, nitrogen, oxygen, silicon, and potassium in untreated, and plasma-treated *Arabidopsis* seeds. From top to bottom, plasma treatment used different times, voltages, and plasma-seed gap distances.

Table S1. Number of NGS-RNA-seq reads before and after quality check on the raw sequencing data for 7-day-old seedlings treated with 7.5 kV plasma.

Sample name	Raw reads	Trimmed reads	Mean GC	Condition
control 1	46625605	45466669	44	control
control 2	46523629	45454673	44	control
control 3	37351249	36364586	44	control
7.5kV_1	47453047	46351468	44	7.5kV
7.5kV_2	44547099	43457657	44	7.5kV
7.5kV_3	49914543	48712288	44	7.5kV

Table S2. Number of clean reads mapped against *A. thaliana* genome for 7-day-old seedlings treated with 7.5 kV plasma

Sample	Input reads	Unique	Multi	Unmapped	Mismatch ratio	Assigned GTF
control 1	45466669	44491934 (97.8562%)	590239 (1.29818%)	384496 (0.845666%)	0.08%	44055690 (96.8967%)
control 2	45454673	44550817 (98.0115%)	631984 (1.39036%)	271872 (0.598117%)	0.08%	43983665 (96.7638%)
control 3	36364586	35579837 (97.842%)	502708 (1.38241%)	282041 (0.775592%)	0.08%	35238242 (96.9026%)
7.5kV_1	46351468	45439262 (98.032%)	601432 (1.29755%)	310774 (0.670473%)	0.08%	44940088 (96.955%)
7.5kV_2	43457657	42405516 (97.5789%)	660139 (1.51904%)	392002 (0.902032%)	0.08%	42010580 (96.6701%)
7.5kV_3	48712288	47418251 (97.3435%)	746002 (1.53145%)	548035 (1.12504%)	0.08%	47118110 (96.7274%)

Table S3. List of DEGs using a 60 s, 7.5 kV plasma treatment with **(left)** upregulated genes and **(right)** downregulated genes with their corresponding fold change.

Upregulated genes			Downregulated genes		
**TAIR ID**	**Gene description**	**Log2FC**	**TAIR ID**	**Gene description**	**Log2FC**
**AT5G62340**	plant invertase/pectin methylesterase inhibitor	**2.143896252**	**AT3G28345**	encodes an ATP-binding cassette (ABC) transporter	**-0.402876008**
**AT1G26240**	proline-rich extensin-like family protein	**1.615083811**	**AT1G66200**	encodes a cytosolic glutamate synthetase	**-0.410588965**
**AT2G43610**	chitinase family protein	**1.413598141**	**AT3G01420**	encodes an alpha-dioxygenase involved in protection against oxidative stress and cell death	**-0.447290809**
**AT1G66270**	BGLU21 encodes a beta-glucosidase that has a high level of activity against the naturally occurring secondary metabolite scopolin.	**1.3941619**	**AT3G13610**	encodes a Fe(II)- and 2-oxoglutarate-dependent dioxygenase family gene F6'H1	**-0.481556848**
**AT1G21890**	nodulin MtN21-like transporter family protein	**1.342477103**	**AT4G01950**	putative sn-glycerol-3-phosphate 2-O-acyltransferase	**-0.488511631**
**AT2G45050**	encodes a member of the GATA factor family of zinc finger transcription factors.	**1.324256008**	**AT5G24030**	encodes a protein with ten predicted transmembrane helices. The SLAH3 protein has similarity to the SLAC1 protein involved in ion homeostasis	**-0.513357358**
**AT2G25980**	mannose-binding lectin superfamily protein	**1.15095165**	**AT4G30280**	encodes a xyloglucan endotransglucosylase/hydrolase	**-0.516919325**
**AT4G15160**	protease inhibitor/seed storage/lipid transfer protein (LTP) family	**1.095722356**	**AT3G12700**	encodes an aspartic protease has an important regulatory function in chloroplasts that not only influences photosynthetic carbon metabolism but also plastid and nuclear gene expression	**-0.519596719**
**AT1G74500**	encodes a basic helix/loop/helix transcription factor that acts downstream of MP in root initiation	**1.094756193**	**AT3G49120**	class III peroxidase Perx34	**-0.525077223**
**AT1G67750**	pectate lyase family protein	**1.035072465**	**AT1G14130**	DAO1 is an IAA oxidase expressed in many different plant parts	**-0.534107928**
**AT2G23050**	a member of the NPY gene family - involved in auxin-mediated organogenesis.	**1.00036748**	**AT1G51420**	sucrose-phosphatase 1	**-0.544192088**
**AT5G19340**	hypothetical protein	**0.894282644**	**AT4G36880**	cysteine proteinase1	**-0.553001556**
**AT3G16390**	encodes a nitrile-specifier protein	**0.868383943**	**AT4G19810**	encodes a Class V chitinase that is a part of glycoside hydrolase family 18 based on CAZy groupings	**-0.569442323**
**AT5G19520**	mechanosensitive channel of small conductance-like	**0.857801365**	**AT5G03355**	cysteine/histidine-rich C1 domain protein	**-0.656741451**
**AT2G34020**	calcium-binding EF-hand family protein	**0.840951663**	**AT1G72140**	tonoplast localized pH dependent, low affinity nitrogen transporter	**-0.667274669**
**AT4G28650**	encodes one of the two putative eLRR kinase closely related to PXY	**0.807505367**	**AT1G21910**	encodes a member of the DREB subfamily A-5 of ERF/AP2 transcription factor family	**-0.687711813**
**AT3G50640**	hypothetical protein	**0.766872726**	**AT5G06720**	encodes a peroxidase with diverse roles in the wound response, flower development, and syncytium formation	**-0.770210545**

**AT3G16410**	encodes a nitrile-specifier protein	**0.765286511**	**AT1G64940**	member of CYP89A	**-0.799393456**
**AT5G62210**	embryo-specific protein 3	**0.764303863**	**AT5G22355**	cysteine/Histidine-rich C1 domain family protein	**-0.824153639**
**AT1G12080**	vacuolar calcium-binding protein-like protein	**0.744265471**	**AT3G56980**	encodes a member of the basic helix-loop-helix transcription factor protein	**-0.848029051**
**AT3G24240**	RGFR1 is a leucine--rich repeat receptor kinase that, together with RGFR2 and RGFR3, binds ROOT GROWTH FACTORS and is required for establishing the gradient of PLETHORA1 and PLETHORA2 essential for proper root growth and development.	**0.712304397**	**AT1G77640**	encodes a member of the DREB subfamily A-5 of ERF/AP2 transcription factor family	**-0.875650026**
**AT1G47600**	encodes a myrosinase	**0.698065241**	**AT4G21680**	encodes a nitrate transporter (NRT1.8)	**-0.957209744**
**AT1G51470**	encodes a myrosinase	**0.672534672**	**AT4G38420**	SKU5 similar 9	**-1.033874204**
**AT4G19030**	an aquaporin	**0.640964946**	**AT5G56080**	encodes a protein with nicotianamine synthase activity	**-1.109636627**
**AT5G15230**	encodes gibberellin-regulated protein GASA4	**0.630414814**	**AT3G24982**	receptor like protein 40	**-1.170528146**
**AT5G02260**	member of Alpha-Expansin Gene Family	**0.589658285**	**AT3G56970**	encodes a member of the basic helix-loop-helix transcription factor family protein	**-1.180905902**
**AT3G12610**	plays a role in DNA-damage repair/toleration	**0.589518673**	**AT3G21500**	encodes a protein that has very high sequence similarity to 1-deoxy-D-xylulose-5-phosphate synthase proteins but does not possess appreciable activity in vitro	**-1.231018665**
			**AT3G02885**	GASA5, is involved in the regulation of seedling thermotolerance.	**-2.488621571**
			**AT5G45890**	senescence-associated gene 12 (SAG12) encoding a cysteine protease influenced by cytokinin, auxin, and sugars.	**-2.490121601**
